# Use of primary health care services among older patients with and without diabetes

**DOI:** 10.1186/s12875-022-01844-2

**Published:** 2022-09-09

**Authors:** Anna-Kaisa Aro, Merja Karjalainen, Miia Tiihonen, Hannu Kautiainen, Juha Saltevo, Maija Haanpää, Pekka Mäntyselkä

**Affiliations:** 1 Insitute of Public Health and Clinical Nutrition, General Practice, University of Eastern Finland, PO Box 1627, FI-70211 Kuopio, Finland; 2 Rantakylä Health Center, Joensuu, Finland; 3 Siilinjärvi Health Center, Siilinjärvi, Finland; 4 School of Pharmacy, University of Eastern Finland, Kuopio, Finland; 5 Unit of Primary Health Care, Helsinki University Central Hospital, Helsinki, Finland; 6 Central Finland Central Hospital, Jyväskylä, Finland; 7 Ilmarinen Mutual Pension Insurance Company, Helsinki, Finland; 8 Primary Health Care Unit, Kuopio University Hospital, Kuopio, Finland

**Keywords:** Type 2 diabetes, Healthcare use, Primary care, Older patients

## Abstract

**Background:**

The aim of this study was to compare the utilization of primary healthcare services by older patients with and without type 2 diabetes.

**Methods:**

Electronic patient records were used to identify persons over 65 years of age with a diagnosis of diabetes. Two age- and sex-adjusted controls without diabetes were extracted for each person with diagnosis of diabetes. A health questionnaire was sent by mail to 527 people with diabetes and 890 controls. Of the persons who answered the questionnaire, 518 persons were randomly selected to participate in a health examination. The study group in this analysis consisted of 187 persons with diabetes and 176 persons without diabetes who attended the health examination. The data on primary health care utilization were extracted from electronic patient records one year before and one after the health examination.

**Results:**

Before the onset of the study, the patients with diabetes had more doctor’s appointments (*p* < 0.001), nurse’s appointments (< 0.001) and laboratory tests taken (*p* < 0.001) than those without diabetes After 1-year follow-up period the patients with diabetes had more doctor’s appointments (*p* = 0.002), nurse’s appointments (*p* = 0.006), laboratory tests taken (*p* = 0.006) and inpatient care at the community hospital (*p* = 0.004) than patients without a diagnosis of type 2 diabetes. The use of the community hospital increased significantly among patients with diabetes (ratio 2.50; 95% Cl 1.16–5.36) but not by patients without diabetes (ratio 0.91; 95% Cl 0.40.2.06). The number of nurse’s appointments increased for patients without diabetes (ratio 1.31; 95% Cl 1.07–1.60) but not for those with diabetes (ratio 1.04; 95% Cl 0.88–1.24).

**Conclusions:**

Patients with diabetes visit more often physicians and nurses compared with those without diabetes. During a 1-year follow-up, the use of community hospital care increased significantly among patients with diabetes. In addition to focusing on prevention and care of diabetes, these results suggest the importance of diabetes in planning community-based health care services.

## Background

Diabetes is a growing healthcare problem worldwide [1]. Diabetes and its treatment impose a great economic burden not only on the society but also on individuals [2]. The costs are anticipated to continue rising in the future as the prevalence of diabetes rises [3]. Drug expenditures and hospital inpatient care form the largest groups of the direct costs [4, 5].

Studies have shown that patients with type 2 diabetes use healthcare services more than people without diabetes. A large proportion of patients with diabetes have at least one comorbid chronic condition [1]. Compared to the general population a diagnosis of type 2 diabetes increases the risk for hospital care [6–9]. Comorbidities, previous hospitalization [10] and poor glycemic control [11] are risk factors for inpatient care in patients with diabetes. Age, insulin use, renal insufficiency and being female have been found to increase the risk of diabetes related-inpatient care [11].

In many countries around the world the proportion of the older people is still increasing during the next decades. At the same time, the prevalence of diabetes is increasing. Most of the studies concerning the use of health care by patients with diabetes are based on hospitals and specialized health care. However, primary health care is responsible for most of the care of these patients with this very common disease. There are some studies focusing on use of the health care at the primary health care level [12–14]. However, there are fewer studies comparing the use of different primary health care services including the use of a primary care -based community hospital between patients with and without diabetes. Therefore, in the present study, we focused on the patterns of service use especially at the primary health care level. The aim of this study was to evaluate at the primary level healthcare use in persons with and without type 2 diabetes aged 65 or older in a primary care setting.

## Methods

### Context

In the Finnish health care system, most primary care services are provided by health centers. Those services include physician’s and nurse’s appointments, laboratory, x-ray and physiotherapy services. In addition to hospital inpatient care, inpatient care is offered in community hospital wards to patients who do not need specialized care [15].

### Study population

This study is a part of the Inner-Savo DM65 + study. The basic population (*N* = 3,093) consisted of home-dwelling people aged at least 65 living in the communities of Suonenjoki and Rautalampi in Eastern Finland. People with a diagnosis of diabetes were identified from primary care electronic patient record according to the International Classifications of Diseases (ICD-10) with diagnostic codes E10 and E11 [16]. People living permanently in institutional care, those who had moved outside the study municipalities and deceased were excluded from the study group. Two age- and sex adjusted controls without diabetes were extracted for each person with a diagnosis of diabetes. A health questionnaire was sent by mail to 527 people with diabetes and 890 controls in 2015 and it was answered by 430 (81.6%) persons with diabetes and 654 (73.5%) by controls. Of the persons who answered the questionnaire, 518 persons were randomly selected and invited to participate in a health examination. The present study population consisted of 359 patients who attended a health examination conducted by one member of the research group (MK) over a period of 3 months in the 2015. Of these, 187 patients had diabetes (of whom five had type 1 diabetes and 182 had type 2 diabetes) and 176 patients had not diabetes. The health examination included a structured interview, a clinical examination and laboratory tests (e.g. fasting plasma glucose and glycated hemoglobin).

### Measurements and tools

In the physical examination the patients’ state of health was evaluated and measured in a standard manner by a doctor. The health examination included measurements of height and weight. Body mass index (BMI) was calculated as weight (kg) / height (m^2^). Blood pressure was measured twice in a sitting position at five-minute intervals after 10 min of rest and average systolic and diastolic pressures were calculated and used as the patient’s blood pressure.

Health-related quality of life (HRQoL) was measured by EuroQol (EQ-5D) [17]. It is a generic measure that includes two parts: a descriptive system and visual analogue scale (EQ VAS). The descriptive system defines health-related quality of life using five dimensions: mobility, self-care, usual activities, pain/discomfort, anxiety/depression.

Self-rated health (SRH) [18] was evaluated by the response to the question: “Is your health in general: excellent, very good, good, fair or poor?” Excellent, very good and good were recorded as good SRH. The Geriatric Depression Scale (GDS-15) [19] was used to evaluate depressive symptoms. Higher scores indicate more symptoms. The Mini-Mental Examination State (MMSE) [20] was used to measure cognitive impairment, with higher scores indicating better cognitive functioning. The Lawton Instrumental Activities of Daily Living (IADL) Scale [21] was used to assess the patient’s ability to function. Higher IADL scores indicate lower functional capacity. Physical activity was measured with the Kasari-FIT index [22]. It includes questions on the frequency, intensity, and duration of exercise. Alcohol consumption was screened by using the Alcohol Use Disorders Identification Test (AUDIT) [23]. Higher scores in this self-reported questionnaire are an indicator of harmful alcohol use.

Comorbidities were recorded by the physician according to a list of the most common chronic diseases. The Charlson comorbidity index [24] was calculated to evaluate the burden of disease (without diabetes) among individuals.

To define the basic health care use before the study, the data were collected from the patient record system during a period of year (12 months) before the health examination. Respectively, to define the health care use prospectively, the data were collected during a period of year after (12 months) the health examination from the patient record system. Doctor’s and nurse’s appointments, dental care and emergency visits, and also impatient care at the community hospital were calculated. The number of laboratory tests and x-ray studies were also calculated.

### Statistical analysis

Descriptive characteristics are presented as means with SDs or as counts The groups were compared using the t test or bootstrap type t test for continuous variables and Chi-square test for categorical variables. The use of primary health care services were analyzed by using Poisson’s model and reported as visits and incidence rate ratios (IRRs) with 95% confidence intervals (CIs). Random-effects Poisson regression models (unstructured correlation structure) were used to evaluate relative changes in health care services [25]. The assumptions of overdispersion in the Poisson models were tested using Lagrange multiplier test. Analyses were adjusted with age, gender, Charlson Index, GDS-15, and physical activity, when appropriate. Collinearity was checked using the variance inflation factor. The bootstrap method, resampling with replacement (10,000 replications), was used when the theoretical distribution of the test statistics was unknown or in the case of violation of the assumptions (e.g. non-normality) [26, 27]. The normality of variables was evaluated graphically and by using the Shapiro–Wilk W test. The Stata 17.0, StataCorp LP (College Station, TX, USA) statistical package was used for the analysis.

## Results

Table 1 shows the mean characteristics of the study group. The patients with diabetes had a higher body mass index (*p* < 0.001), higher GDS-15 scores (*p* < 0.001) and poorer health-related quality of life (*p* < 0.001) than the controls. The patients with diabetes were less likely to smoke (*p* = 0.021) and got lower scores from the AUDIT questionnaire (*p* = 0.013) than the controls. The patients without diabetes were more commonly able to move without assistive aid (*p* < 0.001) and had better physical activity (*p* = 0.03). Patients with a diagnosis of diabetes had more cardiovascular diseases (*p* < 0.001) than patients without diabetes.

**Table 1:** Characteristics of the study patients without diabetes and with diabetes

	Patients without diabetes *N* = 176	Patients with diabetes *N* = 187	*P*-value
Women, n (%)	60(34)	92(49)	0.004
Age, years, mean (SD)	74(6)	74(7)	0.73
Education years, mean (SD)	9.8(3.3)	9.5(3.2)	0.42
Lives alone, n (%)	45(27)	51(30)	0.53
Smoking, n (%)	24(14)	12(6)	0.021
Alcohol use, AUDIT-C, mean (SD)	2.5(2.4)	1.9(2.0)	0.013
Moving without assistive aid, n (%)	147(88)	128(75)	0.003
Physical activity Kasari FIT index, mean (SD)	43.0(21.5)	31.3(20.9)	< 0.001
Depressive symptoms, GDS-15, mean (SD)	2.1(2.4)	3.3(3.1)	< 0.001
Health related quality of life, EQ-5D, mean (SD)	0.829(0.162)	0.757(0.164)	< 0.001
Cognitive status, MMSE, mean (SD)	27.4(3.2)	27.0(3.3)	0.21
Functioning, IADL, mean (SD)	10.9(4.0)	10.8(4.6)	0.84
Good self-rated health, n (%)	97(58)	72(42)	0.004
Body mass index, kg/m^2^, mean (SD)	27.6(5.0)	31.2(5.9)	< 0.001
Fasting plasma glucose, mmol/l, mean (SD)	6.15 (3.75)	7.78 (2.41)	< 0.001
Glycated hemoglobin, mean (SD)	37.4 (3.5)	49.3 (13.6)	< 0.001
Multimorbidity, Charlson Index (diabetes excluded), mean (SD)	0.4(0.8)	0.5(0.8)	0.21
Diseases			
Cardiovascular disease	118(67)	155(83)	< 0.001
Cerebrovascular disease	15(9)	13(7)	0.58
Musculoskeletal diseases	64(36)	73(39)	0.60
Astma or chronic obstructive pulmonary disease	17(10)	19(10)	0.87
Neurological disease	3(2)	7(4)	0.34
Cancer	14(8)	13(7)	0.72
Mental illness	3(2)	8(4)	0.22
Dementia	2(1)	8(4)	0.11

*SD* Standard deviation, *AUDIT-C* The Alcohol Use Disorders Identification Test, *GDS-15* Geriatric Depression Scale, *EQ-5D* EuroQol Questionnaire, *MMSE* Mini-Mental State Examination, *IADL* Lawton Instrumental Activities of Daily Living Scale

The results of this study show that before the onset of the study (Table 2) the patients with diabetes had more doctor’s appointments (*p* < 0.001), nurse’s appointments (< 0.001) and laboratory tests taken (*p* < 0.001) than the controls. These results remained statistically significant even after adjusting for age, gender, Charlson Index, GDS-15 and the patient’s physical activity.

**Table 2:** Use of primary health care services in patients without and with diabetes during the one-year period before the onset of study

	Patients without diabetes *N* = 176	Patients with diabetes *N* = 187	IRR (95% CI)	
	Mean (SE)	Mean (SE)	Crude	Adjusted*
Doctor’s appointment	2.20(0.17)	3.45(0.22)	1.57 (1.29 to 1.91)a	1.50 (1.22 to 1.83)a
Emergency visits	0.09(0.02)	0.13(0.03)	1.41 (0.75 to 2.66)	1.43 (0.71 to 2.85)
Nurse’s appointment	2.64(0.34)	4.97(0.56)	1.89 (1.35 to 2.63)a	1.84 (1.29 to 2.63)a
X-ray studies	0.44(0.06)	0.67(0.08)	1.54 (1.08 to 2.20)b	1.46 (0.98 to 2.12)
Laboratory tests	3.11(0.34)	5.67(0.52)	1.82 (1.38 to 2.41)a	1.82 (1.34 to 2.47)a
Inpatient care	0.76(0.26)	0.97(0.35)	1.28 (0.48 to 3.42)	1.21 (0.40 to 3.64)

*Abbreviation: SE* Standard error, *IRR* Incidence rate ratio

^*^Adjusted for age, gender, Charlson Index, GDS-15, and physical activity

a *p* < 0.001

b *p* = 0.017

After a 1-year follow-up period (Table 3) the patients with diabetes had more doctor’s appointments (*p* = 0.002), nurse’s appointments (*p* = 0.006), laboratory tests taken (*p* = 0.006) and inpatient care at the community hospital (*p* = 0.004) than patients without a diagnosis of type 2 diabetes. These results remained statistically significant even after adjusting for age, gender, Charlson Index (Diabetes mellitus excluded), GDS-15 and patients’ physical activity. Glycated hemoglobin (HbA1c) level did not correlate with the primary health care service use.

**Table 3:** Use of primary health care services in patients without and with diabetes during the one-year follow-up period after the onset of study

	Patients without diabetes *N* = 176	Patients with diabetes *N* = 187	IRR (95% CI)	
	Mean (SE)	Mean (SE)	Crude	Adjusted^a^
Doctor’s appointment	2.30(0.18)	3.16(0.21)	1.38 (1.12 to 1.69) *p* = 0.002	1.33 (1.08 to 1.64) *p* = 0.007
Emergency visits	0.11(0.03)	0.18(0.04)	1.60 (0.88 to 2.92)	1.50 (0.81 to 2.78)
Nurse’s appointment	3.45(0.39)	5.17(0.48)	1.50 (1.12 to 2.00) *p* = 0.006	1.61 (1.17 to 2.20) *p* = 0.003
X-ray studies	0.55(0.06)	0.69(0.07)	1.25 (0.92 to 1.70)	1.34 (0.96 to 1.87)
Laboratory tests	3.32(0.38)	4.94(0.44)	1.49 (1.12 to 1.98) *p* = 0.006	1.43 (1.12 to 1.84) *p* = 0.005
Inpatient care	0.69(0.22)	2.42(0.71)	3.52 (1.49 to 8.30) *p* = 0.004	3.21 (1.26 to 8.16) *p* = 0.014

*Abbreviation: SE* Standard error, *IRR* Incidence rate ratio

^a^ Adjusted for age, gender, Charlson Index, GDS-15, and physical activity

**Fig. 1:**
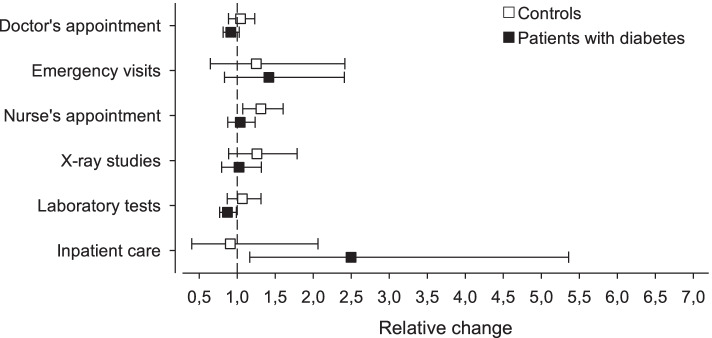
The relative changes (adjusted with age, gender, Charlson Index, GDS-15, and physical activity) in the use of primary health care services within the groups representing patients without diabetes and with diabetes. The change has been calculated from the one-year period before the onset of study to one-year period after the onset of study. Whiskers represents 95% confidence intervals

The biggest relative change from the one-year period before the onset of study to one-year period after the onset of study occurred in inpatient care at the community hospital (Fig. 1). The patients with diabetes had 2.5 times more inpatient care compared to the start of the study whereas there was no change in the control group. The number of nurse’s visits increased slightly in the control group but not among the patients with diabetes.

## Discussion

The purpose of this study was to evaluate the use of different primary healthcare services among older patients with and without diabetes. The results of this study show that patients with diagnosis of diabetes use more healthcare services when it comes to doctor’s appointments, nurse’s appointments, and laboratory tests. After a 1-year follow up, use of primary care-based community hospital increased in patients with diabetes but not in those without diabetes.

Most studies concerning the use of health care services have been more focused on hospital care than to primary health care. They have shown that patients with diabetes have more physician office visits, more outpatient visits and more hospital stays than patients without diabetes. Also, the time spent in hospital is longer compared to patients without a diagnosis of diabetes [2, 4, 9, 28, 29]. In studies by the American Diabetes Association [2], diabetes-related health care use is found to be highest among patients over 65 years of age. On the other hand, some of the previous studies have shown that the risk for hospitalization is more evident in younger age groups of patients with diabetes than with patients over 65-years [30]. Some studies have shown that the use of the emergency department is higher in patients with diabetes [31, 32] but when multivariate models are used the difference is no longer significantly higher [33].

Patients with a diagnosis of diabetes had more doctor’s appointment and laboratory tests taken than patients without diabetes at the onset of the study. This result can be explained by the fact that the treatment of diabetes includes checkup appointments and routine laboratory tests. The number of visits to nurse increased in patients without diabetes but not with diabetes. We assume that this is due to the increased need for follow-up visits due to chronic diseases with increasing age. Among the patients with diabetes, the follow-up visits are better organized and planned to result in that among them there was not a need to increase nurse visits. Usually, the treatment of diabetes is arranged well in the Finnish primary health care and the follow-up of patients is planned [34]. This can result in the more structured and frequent follow-up of patients with diabetes compared with those patients without diabetes which may partly explain these findings. However, it does not explain the increased use of primary care hospital which may be more due to the progress of diseases and the increased multimorbidity burden after the study baseline. It has been found that frequent GP and nurses follow-up may reduce the avoidable use of hospital care. Although the follow-up and monitoring of diabetes seems to be at good level in Finland, we must consider how it could be even improved. Patients with diabetes usually have other diseases for example cardiovascular diseases than those without diabetes resulting an increased risk of hospitalization. In addition, older people with diabetes may be more prone to acute diseases (e.g. infections) and the risk of diabetes-related complications may increase with age. Therefore, management and careful follow-up according to a comprehensive care plan is especially important. In addition, it seems evident that also community hospital plays an important role supporting the primary health care near patients.

It is possible that in addition to diabetes and co-existing health problems there have been other factors potentially explaining our results. Increased multimorbidity among patients with diabetes could be one factor. Although cardiovascular diseases were more common in patients with diabetes as expected, the morbidity burden measured with Charlson Index was not bigger in patients with diabetes than those without. It could be hypothesized that socioeconomic status may be related to increased level of health care use among patients with diabetes. A previous Finnish study found that socioeconomic status did not associate with less favorable results of lifestyle counselling [35]. A recent primary care registry-based study found that socioeconomic status did not associate with the delivery of care among patients with diabetes [36]. In our study, we were able to use two indicators related to socioeconomic status, education and the proportion of those who lived alone. There were not differences between patients with and without diabetes concerning these variables. Therefore, we assume that socioeconomic status did not explain the difference between older patients with and without diabetes concerning health care service use in the present study.

In Finland, the diabetes care is organized according to guidelines which most often means the good treatment situation. It may also mean the more structured and frequent follow-up of patients with diabetes compared with those patients without diabetes. This may partly explain the use of services among patients with diabetes. However, it does not explain the increased use of community hospital which may be more due to the progress of diseases and increased multimorbidity. It has been found that frequent GP and nurse follow-up visits may reduce the avoidable use of hospital care [37].

The examined patients were detected from a population representing older people with the established diagnosis of diabetes and the comparison group of patients without the diagnosis of diabetes. The characteristics of these patients were close to a previous Finnish primary care setting study [38]. The patient population was based on electronic patient records having further collection of data with a questionnaire and a health examination. Although the patients seemed to represent quite well Finnish older primary care patients, they came from one primary care district. Therefore, we must be cautious in interpreting these results more generally. Another limitation is that we did not have exact data on the complications of diabetes. However, in patients with diabetes, the functional capacity and disease burden in general seemed to be like in those patients having not diabetes. The level of glycated hemoglobin (HbA1c) in the present study indicated good treatment balance in general. According to our previous analysis, most of these patients with diabetes had good or at least moderate treatment situation [39]. In addition, there was not significant difference in renal function between patients with and without diabetes [40]. Hence, we can assume that the present study patients with diabetes represent those patients who are able to manage at home.

The results of this study showed that the use of primary care based inpatient care in the community hospital increased clearly after the follow-up but in primary care patients without diabetes the rate of community hospital care did not change. It can be assumed that most of the acute diseases or deterioration of chronic diseases of older people are treated in primary care-based community hospitals in Finland, which has about 200 general practitioner run community hospitals [12]. Diabetes is associated especially with the higher use of specialized care. However, the use of primary health care services including inpatient care in community hospital has not studied as intensively. Therefore, the present study provides new knowledge from that perspective. Our results indicate that the increasing prevalence of diabetes with increasing numbers of older people is related to an increased need for these kinds of low-threshold care facilities including community hospitals outside of specialized health care.

## Conclusions

The results of this primary health care setting study showed especially use of primary care-based hospital care increased during the follow-up period in older patients with diabetes. The population will become older in the next decades and the prevalence of diabetes will increase. Therefore, these results should be considered in regional and national planning of health care and in resource allocation.

## Data Availability

The datasets generated and analysed during the current study are not publicly available due to protection of individual privacy, but are available from the corresponding author on reasonable request.

## Abbreviations

AUDIT: The Alcohol Use Disoreders Identification Test
BMI: Body Mass Index
EQ-5D: EuroQol
GDS-15: The geriatric Depression Scale
HRQoL: Health-related Quaitys of Life
IADL: The Lawton Intrumental Activities of Daily Living
ICD-10: International Classifications of Diseases
MMSE: The Mini-mental Examination State
SHR: Self-rated Health
